# Physical Activity Assessment in Patients with Axial Spondyloarthritis Compared to Healthy Controls: A Technology-Based Approach

**DOI:** 10.1371/journal.pone.0085309

**Published:** 2014-02-28

**Authors:** Thijs Willem Swinnen, Tineke Scheers, Johan Lefevre, Wim Dankaerts, Rene Westhovens, Kurt de Vlam

**Affiliations:** 1 Rheumatology, University Hospitals Leuven, Leuven, Belgium; 2 Department of Development and Regeneration, KU Leuven, Leuven, Belgium; 3 Department of Rehabilitation Sciences, KU Leuven, Heverlee, Belgium; 4 Department of Kinesiology, KU Leuven, Heverlee, Belgium; 5 Research Foundation Flanders, Brussel, Belgium; University of Texas Health Science Center at Houston, United States of America

## Abstract

**Introduction:**

Traditionally, assessment in axial Spondyloarthritis (aSpA) includes the evaluation of the capacity to execute tasks, conceptualized as physical function. The role of physical activity, defined as movement-related energy expenditure, is largely unknown and almost exclusively studied using patient-reported outcome measures. The aims of this observational cross-sectional study are to compare physical activity between patients with aSpA and healthy controls (HC) and to evaluate the contribution of disease activity to physical activity differences between groups.

**Methods:**

Forty patients with aSpA were matched by age, gender, period of data acquisition in terms of days and season to 40 HC. Physical activity was measured during five consecutive days (three weekdays and two weekend days) using ambulatory monitoring (SenseWear Armband). Self-reported disease activity was measured by the Bath Ankylosing Spondylitis Disease Activity Index (BASDAI). Differences in physical activity between patients with aSpA and HC were examined with Wilcoxon signed-rank tests and a mixed linear model. Difference scores between patients and HC were correlated with disease activity.

**Results:**

Average weekly physical activity level (Med(IQR); HC:1.54(1.41–1.73); aSpA:1.45(1.31–1.67),MET) and energy expenditure (HC:36.40(33.43–41.01); aSpA:34.55(31.08–39.41),MET.hrs/day) were significantly lower in patients with aSpA. Analyses across intensity levels revealed no significant differences between groups for inactivity and time spent at light or moderate physical activities. In contrast, weekly averages of vigorous (HC:4.02(1.20–12.60); aSpA:0.00(0.00–1.20),min/d), very vigorous physical activities (HC0.00(0.00–1.08); aSpA:0.00(0.00–0.00),mind/d) and moderate/(very)vigorous combined (HC2.41(1.62–3.48); aSpA:1.63(1.20–2.82),hrs/d) were significantly lower in patients with aSpA. Disease activity did not interact with differences in physical activity between patients with aSpA and HC, evidenced by non-significant and very low correlations (range: −0.06–0.17) between BASDAI and HC-aSpA patients' difference scores.

**Conclusions:**

Patients with aSpA exhibit lower physical activity compared to HC and these differences are independent of self-reported disease activity. Further research on PA in patients with aSpA should be prioritized.

## Introduction

The concept of spondyloarthritis embodies a family of rheumatic diseases characterized by distinct processes of tissue inflammation, destruction and/or pathological bone formation. Articular features typically occur at the synovio-entheseal complex [1], [2], but also extra-articular features such as uveitis and psoriasis may complicate disease [3]. Clinically, a predominantly axial or peripheral articular presentation or a combination of both subtypes can be distinguished [4], [5]. In axial spondyloarthritis (aSpA), inflammatory back pain, stiffness and mobility impairment contribute to limitations in activities and restrictions in societal participation [4], [6].

Physical activity (PA) can be defined as any bodily movement produced by contraction of skeletal muscle that substantially increases energy expenditure [7]. Community-based PA interventions for people with arthritis in general have shown to improve physical function, decrease pain, delay functional decline and reduce costs [8], [9]. Despite this ample interest in PA in patients with arthritis in general, PA is a neglected construct in the aSpA literature [10]. The Assessment of SpondyloArthritis international Society (ASAS) expert group and the European League Against Rheumatism recommended exercise, a structured and planned form of PA [7], as a decisive part of the non-pharmacological treatment of ankylosing spondylitis (AS), [11]. Exercise programs for patients with AS, the hallmark aSpA condition, traditionally include flexibility exercises with only minor benefits on physical function, spinal mobility and patient global assessment at best [12], [13]. Typically, these programs fail to deliver the optimal PA intensity according to the American College of Sports Medicine (ACSM) recommendations to develop health-related physical fitness in terms of cardio-respiratory endurance, muscular strength and body composition [12]. In contrast to other arthritis subgroups such as rheumatoid arthritis and osteoarthritis, the efficacy and safety of PA in relation to health outcomes is unknown for aSpA. Limited evidence from cross-sectional studies indicates a role for PA to improve fatigue [14], body composition [15] and quality of life [16], similar to findings in the healthy population. However, if patients exhibit less PA [15]–[17] and different i.e. disease-specific PA patterns compared to healthy controls is largely unknown. These data are needed to guide health policy and set research priorities.

PA assessment is currently not included in the ASAS minimal core set to monitor patients with aSpA in both clinical practice and research [18]. The key domain ‘physical function’ reflects difficulties in executing physical activities, not their amount or intensity, and is evaluated with the self-reported Bath Ankylosing Spondylitis Functional Index [19], [20]. Since both expert rheumatologists and rehabilitation experts in the aSpA field increasingly recognize the need to establish the possible dose-dependent effects of PA [12], [16], [21], novel PA assessment strategies such as accelerometry are needed. Further, low correlations between PA and physical function measures in rheumatoid arthritis [22] or osteoarthritis [23] indicate that these related but distinct concepts should be assessed separately to optimally describe functioning. Taken together, establishing the role of PA in aSpA may lead to new perspectives on both the assessment of functioning and efficacy of PA on several clinical outcomes. This study investigated the role of self-reported disease activity in explaining PA differences.

To our best knowledge, this is the first study that aimed to identify differences in weekly PA between patients with aSpA and healthy controls using objective monitoring of PA in free-living conditions with a sophisticated multi-sensor device. Additionally, between and within group differences in PA will be explored for each timepoint (weekdays, Saturday and Sunday) to further detect different PA patterns. Lastly, this study aimed to unravel the role of disease activity in explaining the observed differences in PA between patients with aSpA and healthy controls.

## Materials and Methods

### Subjects

Forty patients with aSpA were consecutively recruited from our spondyloarthritis outpatient clinic at the University Hospitals Leuven. Axial SpA diagnosis was verified by an expert rheumatologist according to the European Spondylarthropathy Study Group criteria [5]. Exclusion criteria were: 1) history of spinal fractures or other fractures within 12 months, lower quadrant musculoskeletal injuries not related to SpA, discitis, pregnancy, spondylolisthesis, spondylolysis, 2) current symptoms of severe health conditions (eg. heart failure) that would influence the PA assessment according to the principal investigator, 3) not being able to stand or walk without an aid. An experienced physical therapist ascertained exclusion criteria using the patient's medical record and the Self-administered Co-morbidity Questionnaire [24]. Forty healthy controls, matched by gender, age and period of data acquisition (season and monitoring days), were randomly selected from a large study on PA in Flemish adults [25]. A random number calculator (www.randomization.com) guided the selection procedure within strata of possible matches. All subjects provided written informed consent prior to participation. The study protocol was written in accordance to the Declaration of Helsinki and was approved by the Medical Ethics Committee of the University Hospitals Leuven (ML 5236).

### Measurements

#### Disease activity

The Bath Ankylosing Spondylitis Disease Activity Index (BASDAI), originally developed in patients with AS, is the widely accepted and ASAS endorsed disease-specific instrument to assess disease activity in aSpA [26]. The BASDAI questionnaire comprises six questions to evaluate the severity of fatigue, peripheral and axial pain, localized tenderness and morning stiffness during the last week. The psychometric properties of the BASDAI are well established [27]–[29].

#### Physical activity

The SenseWear Pro 3 Armband (BodyMedia, Pittsburgh, USA) is a multi-sensor device containing a two-axial accelerometer and sensors measuring heat flux, galvanic skin response, skin temperature and near-body ambient temperature. The armband is positioned over the triceps muscle of the right arm. Algorithms provided by the manufacturer combine the sensor data with age, body weight, height, gender, smoking status and handedness to produce minute-by-minute estimates of energy expenditure (kcal), physical activity intensity (metabolic equivalent) and number of steps. Axial SpA patients and healthy controls were instructed to continuously wear the Armband for 5 and 7 consecutive days respectively, except during water-based activities which were reported in a non-wear log. A valid day was defined as a wear time of minimally 1296 minutes, which corresponds to 90% of a 24 hour period. To avoid bias, we selected the same weekdays in patients and healthy controls. Anthropometric measures were taken by the same observer at the moment of the outpatient visit, prior to the monitoring period. Height was measured with a stadiometer (Holtain Ltd, Dyfed, UK) to the nearest 0,1 cm and weight was measured with a digital scale (SECA, Birmingham, UK) to the nearest 0,1 kg. PA parameters were calculated for weekdays (average of three weekdays), Saturday and Sunday. Furthermore, a weekly average was estimated by the formula: ((parameter_average weekday_ * 5)+parameter_Saturday_+parameter_Sunday_)/7. Physical activity level (PAL) and energy expenditure (EE) both reflected the average daily energy expenditure, expressed as a multiple of the resting metabolic rate of 1 metabolic equivalent (1 MET = 1 kcal/kg/hr) and in MET.hrs/d, respectively. Time spent at different PA intensity levels was obtained using MET-values. MET-values ≤1.8 were considered to reflect inactivity, whereas MET-values >1.8 and <3 were defined as light activity [30]. MET-values ≥3 and <6 were classified as moderate activities. Vigorous activities were characterised by MET-values ≥6, but <9. MET-values ≥9 indicated very vigorous activities [31]. MET-values ≥3 reflected the overall health enhancing moderate and (very)vigorous physical activity (MVPA) estimate. The validity of the SenseWear in assessing these PA parameters is established in both healthy [32] and diseased persons [33].

### Data analysis

Continuous descriptive data of patients and healthy controls were contrasted using a paired t-test to account for the matched nature of the study and Chi-squared tests for proportions (p<0.05). The *primary outcome analyses* involved: 1) the comparison of weekly average PA parameters between aSpA patients and controls using a Wilcoxon signed-rank test and 2) the verification of an interaction effect of disease activity on all PA comparisons between patients with aSpA and healthy controls. Difference scores within each matched pair (controls minus patients) were calculated and correlated with disease activity (BASDAI) with Spearman rank correlation coefficients. Because of the typical and large difference in work status between groups that may explain observed PA inequalities, we also correlated work status with PA difference scores using the point biserial correlation coefficient (spearman rank coefficient yielded the same results). A significant (p<0.05) and moderate (>0.30) coefficient was a priori set as the threshold for an interaction effect [34]. The *secondary outcome analyses* were exploratory comparisons (no a priori power calculations for this part) of PA parameters 1) between groups at any of the individual timepoints namely weekdays, Saturday and Sunday, 2) within each group across all timepoints and 3) between groups to detect different change patterns in PA estimates across all timepoints. All comparisons required longitudinal analyses whereby both the timepoints and groups were regarded as repeated measures. A general linear model which models covariances was employed using the MIXED procedure in SAS version 9.3 (SAS Institute, Cary, USA). For both timepoints and group, an unstructured covariance matrix was assumed. The model included fixed effects for time, group and their interaction. Model assumptions of constant variance and normality of the residuals were assessed by visual inspection of residual plots. Log-transformation was applied to PAL and EE to correct distorted residuals. However, log-transformation still yielded inappropriate residuals for time spent at light, moderate, vigorous, very vigorous and MVPA. For these parameters, the first and third types of comparison were made by means of a Friedman's test. To assess whether there was a difference between the groups at any time, a generalized estimating equations (GEE) model with identify link and normal distribution for the residuals was employed using sandwich estimators for the estimation of the (co)variances since it has been shown that this analysis yields consistent results, even if the model assumptions have not been satisfied. Post-hoc tests pairwise comparisons were made using Wilcoxon signed-rank tests. In order to attain a probability of 95%, p-values were Bonferroni corrected by multiplying them by 3.

## Results

Demographics and disease related characteristics are presented in Table 1 and indicate successful matching. During non-wear time, neither patients nor healthy controls reported additional PA. Full data are given in Table S1 available on the PLOS ONE website.

**Table 1:** Demographics of healthy controls and patients with axial spondyloarthritis (aSpA).

		Healthy controls n = 40	aSpA patients n = 40	p-value
Gender	Men (n (%))	24 (60%)	24 (60%)	NA
	Women (n (%))	16 (40%)	16 (40%)	NA
Work status (n with job (%))		39 (98%)	25 (63%)	<.001$
Weight (kg)		75.69±13.31	76.36±17.12	.847¶
Height (cm)		173.63±9.75	170.15±10.13	.121¶
Body Mass Index (kg/m^2^)		25.05±3.59	26.27±5.11	.219¶
Age (years)		44.33±10.63	44.38±11.30	.984¶
SWA wear-time (hrs/d)		23.71±0.17	23.67±0.03	.501¶
Disease duration (years)		NA	11.40±9.50	NA
BASDAI (0–10)		NA	3.69±2.59	NA
Peripheral joints (0–10)*		NA	3.10±2.97	NA
BASFI		NA	3.52±2.50	NA
Cervical rotation (°)#		NA	62.41±14.61	NA
Tragus to wall distance (cm)#		NA	13.23±3.73	NA
Chest expansion (cm)		NA	4.06±1.98	NA
Lumbar side flexion#		NA	11.23±4.09	NA
Modified Schöber Index (cm)#		NA	3.59±1.00	NA
Intermalleolar distance (cm)#		NA	97.43±20.00	NA
BASMI (0–10)		NA	3.05±1.21	NA
TSK-AA (11–44)		NA	13.83±3.28	NA
NSAIDs (n (%))		NA	21 (52,5%)	NA
Biologicals (n (%))		NA	19 (47,5%)	NA
Analgesics (n (%))		NA	14 (35%)	NA
DMARDs (n (%))		NA	8 (20%)	NA
Corticosteroids (n (%))		NA	0 (0%)	NA
Psychopharmaca (n (%))		NA	3 (7,5%)	NA

BASDAI, Bath Ankylosing Spondylitis Disease Activity Index; BASFI, Bath Ankylosing Spondylitis Functional Index; BASMI, Bath Ankylosing Spondylitis Metrology Index; NSAIDs, Non-Steroidal Anti-Inflammatory Drugs; DMARDs, Disease-Modifying AntiRheumatic Drugs; SWA, SenseWear Armband; TSK-AA, Tampa Scale for Kinesiophobia Activity Avoidance subscale;

*item 3 BASDAI;

# based on BASMI;

¶ paired t-test (p<.05);

$ chi-square test (p<.05);

NA, not applicable.

### Weekly physical activity between groups

**Figure 1:**
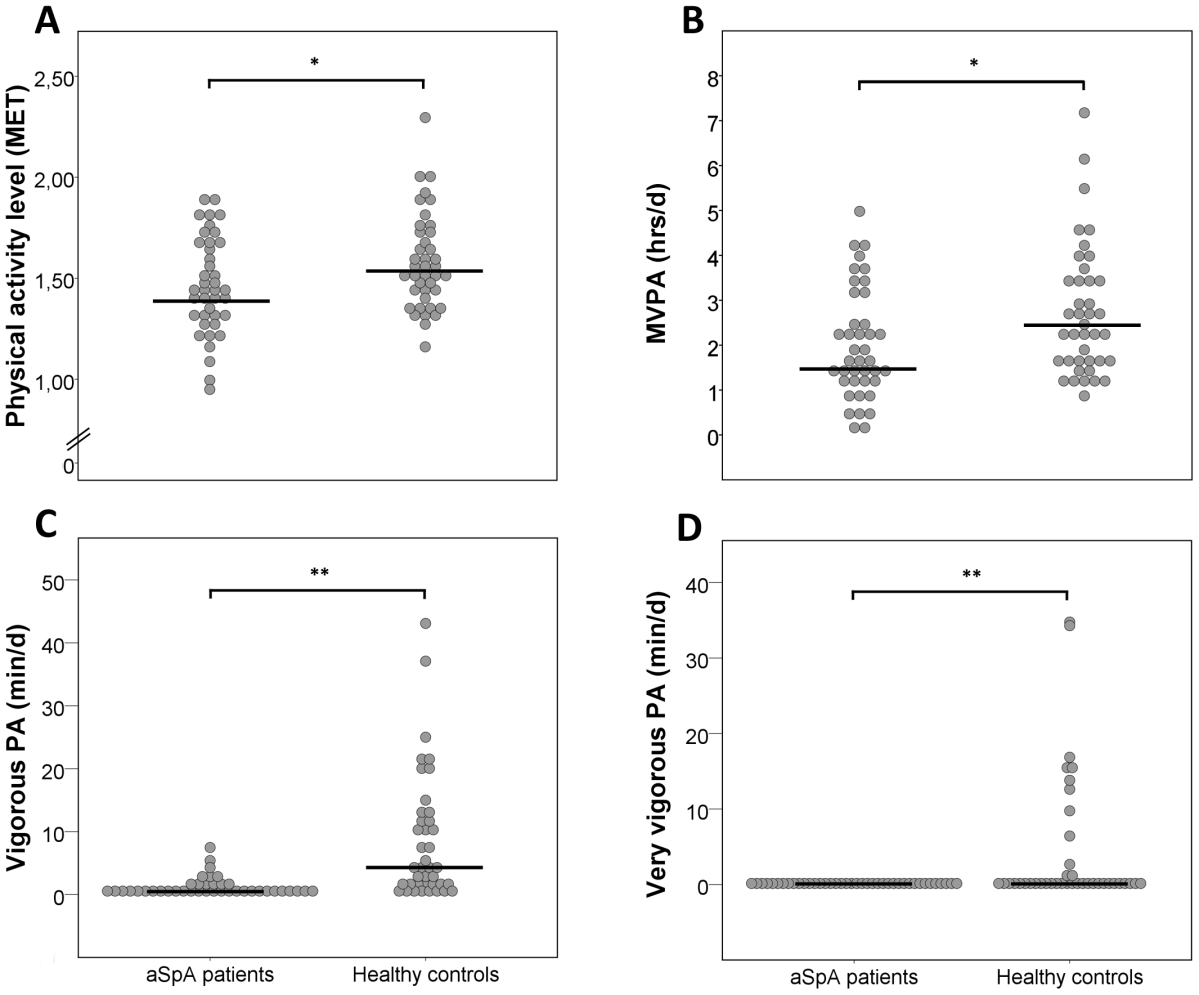
Individual physical activity data between controls (n = 40) and patients with axial spondyloarthritis (n = 40): physical activity level expressed in metabolic equivalent (MET) (A), time spent at moderate and (very)vigorous physical activity (MVPA) in hrs/d (B), time spent at vigorous (C) and very vigorous (D) physical activities in min/d; *p<0.05, **p<0.01.

Results of our primary outcome analysis are given in Table 2. Weekly PAL (p = 0.048) and EE (p = 0.045) were significantly lower in patients with aSpA (Table 2, Figure 1A). No differences between groups were found for weekly time spent at inactivity, light or moderate PA. For the latter, a trend for less moderate PA in patients was observed (p = 0.07). A lack of vigorous (p<0.001) and very vigorous (p<0.001) weekly PA in patients with aSpA versus controls was detected (Figure 1C, 1D, 3), in addition to reduced levels of MVPA combined (p = 0.029; Table 2, Figure 1B).

**Figure 2:**
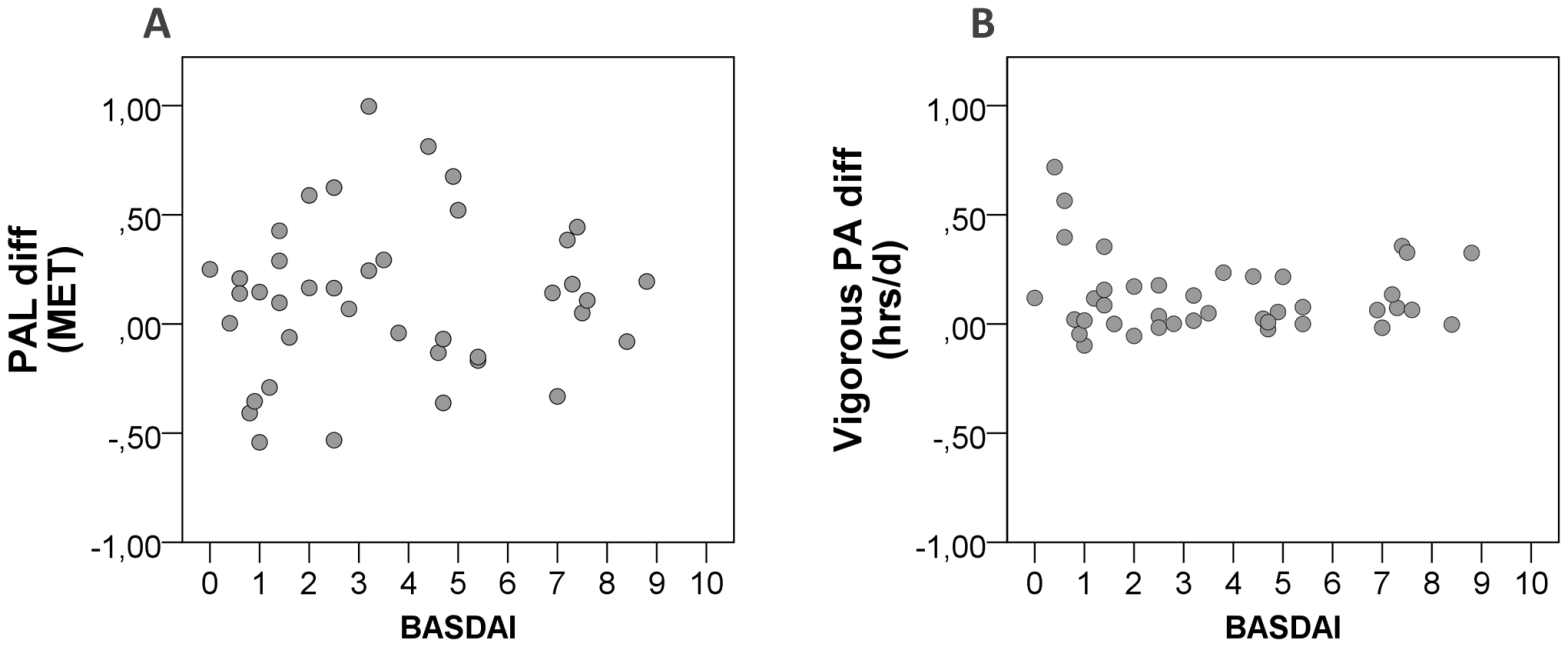
Scatterplots of healthy control versus axial spondyloarthritis (aSpA) difference scores and disease activity as measured by the Bath Ankylosing Spondylitis Disease Activity Index (BASDAI): Physical activity level (PAL) A) and Vigorous PA (B); PA, physical activity; MET, metabolic equivalent; diff, difference score: for each matched pair (n = 40 pairs) healthy control value minus aSpA patient value.

**Figure 3:**
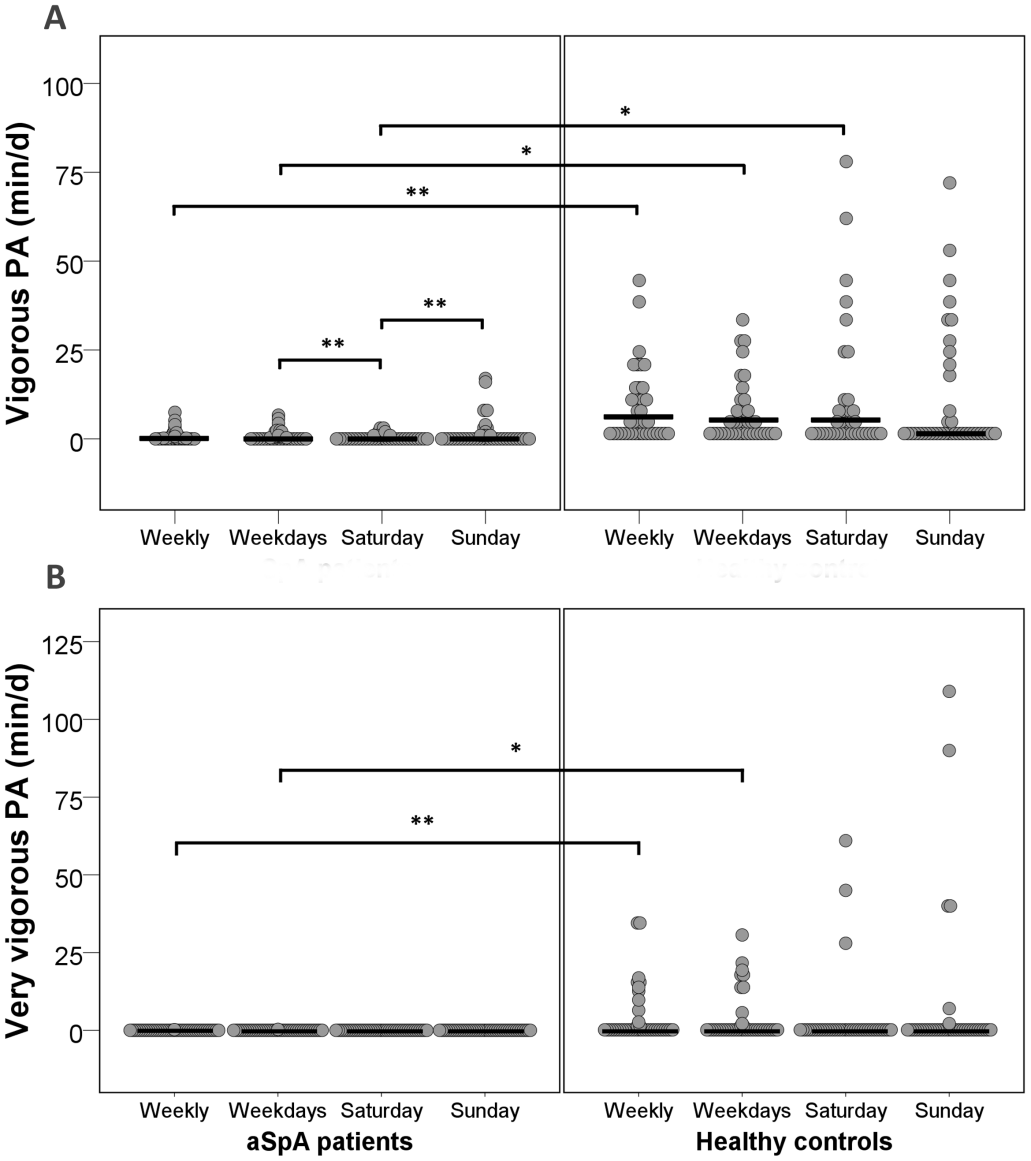
Vigorous (A) and very vigorous (B) physical activities (PA) expressed in min/d for patients with axial spondyloarthritis (aSpA, n = 40) and matched healthy controls (n = 40). Horizontal lines represent median values for weekly average and time point estimates; *p<0.05, **p<0.01.

**Table 2:** Comparison of physical activity parameters between healthy controls and patients with axial spondyloarthritis (aSpA).

		Healthy controls (n = 40)#	aSpA patients (n = 40)#	p-value¶
Weekly averages*				
PAL	(MET)	1.54 (1.41–1.73)	1.45 (1.31–1.67)	**.048**
EE	(MET.hrs/d)	36.40 (33.43–41.01)	34.55 (31.08–39.41)	**.045**
Inactive	(hrs/d)	17.85 (16.44–18.95)	17.99 (16.83–19.17)	.450
Light PA	(hrs/d)	3.28 (2.73–4.10)	3.87 (2.73–4.48)	.288
Moderate PA	(hrs/d)	2.29 (1.53–3.22)	1.63 (1.20–2.80)	.070
	(min/d)**	137.40 (91.80–193.20)	97.80 (72.00–168.00)	.070
Vigorous PA	(hrs/d)	0.07 (0.02–0.21)	0.00 (0.00–0.02)	**<.001**
	(min/d)**	4.02 (1.20–12.60)	0.00 (0.00–1.20)	**<.001**
Very vigorous PA	(hrs/d)	0.00 (0.00–0.03)	0.00 (0.00–0.00)	**<.001**
	(min/d)**	0.00 (0.00–1.08)	0.00 (0.00–0.00)	**<.001**
MVPA	(hrs/d)	2.41 (1.62–3.48)	1.63 (1.20–2.82)	.**029**
	(min/d)**	144.71 (96.98–208.05)	98.19 (71.93–169.26)	.**029**

# Data are presented as median (quartile range); PAL, physical activity level; EE, energy expenditure; PA, physical activity; MVPA, moderate/(very)vigorous physical activity combined;

*for a total week estimate, multiply values with seven;

**estimates transformed to minutes to facilitate interpretation;

¶ Wilcoxon signed-rank tests, significant results in bold, p<0.05.

### Role of disease activity and work status

Disease activity did not interact with differences in weekly PA between patients and controls, evidenced by non-significant and very low correlations between BASDAI and difference scores ranging from −0.06 to 0.17 (Table 3, Figure 2). Similarly low and non-significant correlations between work status and difference scores were observed ranging from −0.11 to 0.12 (detailed data available from the corresponding author upon request).

**Table 3:** Spearman correlation coefficients between disease activity (BASDAI*) and difference scores between healthy controls (HC) and patients with axial spondyloarthritis (aSpA) for all weekly average physical activity parameters (n = 40).

	HC-aSpA patients difference scores#	R¶	p-value
Weekly averages**			
PAL (MET)	0.14 (0.40)	0.07	.656
EE (MET.hrs/d)	3.15 (9.64)	0.08	.605
Inactivity (hrs/d)	−0.25 (2.87)	−0.02	.881
Light PA (hrs/d)	−0.49 (2.32)	−0.06	.710
Moderate PA (hrs/d)	0.43 (3.33)	0.17	.300
Vigorous PA (hrs/d)	0.07 (0.19)	−0.04	.815
Very vigorous PA (hrs/d)	0.00 (0.03)	0.06	.706
MVPA (hrs/d)	0.66 (2.09)	0.12	.452

# Data are presented as median (quartile range);

*BASDAI, Bath Ankylosing Spondylitis Disease Activity Index; PA, physical activity; PAL, physical activity level; EE, energy expenditure; MVPA, moderate/(very)vigorous physical activity combined;

**for a total week estimate, multiply values with seven;

¶ Correlation coefficients were neither significant nor relevant, no interaction was observed, p<0.05.

### Differences between groups at time points

Concerning the secondary outcome analyses, significant differences between groups at any time point were found only for vigorous (p<0.001), very vigorous PA (p = 0.015) and MVPA (p = 0.028) (see Table S1). Patients were spending less time at vigorous PA on weekdays (p<0.001) and Saturday (Figure 3A, p<0.001). Significantly less very vigorous PA on weekdays (Figure 3B, p = 0.015) and less MVPA on Saturday (p = 0.021) was found in patients. All plots have shown a pattern of less PA in aSpA patients at each time point (Figures not shown, additional figures available upon request from the corresponding author).

### Differences within groups at timepoints

Several significant effects within the aSpA group were detected (Table S1, EE: p = 0.039, light PA: p = 0.049; vigorous PA: p = 0.009). The visually clear lower EE on Sunday compared to Saturday (p = 0.097) and increased MVPA on Saturday (p = 0.397) did not reach significance. In contrast, patients with aSpA were showing significantly more time spent at light PA on weekdays compared to Sunday (p = 0.021). Also, patients were spending significantly more time at vigorous PA (Figure 3A) on weekdays compared to Saturday (p = 0.027), but not on Saturday compared to Sunday (p = 0.128). Significant effects within the healthy control group were identified for time spent inactive (p = 0.013) and at light PA (p = 0.020), with less inactive time on Saturday compared to Sunday (p = 0.012) and with more light PA on weekdays than Sunday (p = 0.004).

### Change profile across timepoints between groups

No significant differences in overall within group change patterns between patients with aSpA and controls were found (p>0.05, Table S1). Visual inspection of all graphs (Figures not shown, but available on request from the corresponding author) has revealed quite stable PA estimates in aSpA patients across timepoints, while more variability on Saturday in the healthy control group was reflected both in the plots and quartile ranges (Table S1).

## Discussion

This is the first study demonstrating differences in PA between patients with aSpA and healthy controls using technology-based PA assessment. The lower weekly average estimates of PAL and EE observed indicate that total PA is reduced in patients with aSpA. To date, only three studies compared total PA between patients with aSpA and healthy controls. Marcora [17] studied disease-related cachexia in 19 patients with AS and 19 age-matched controls. To exclude PA behaviour as a non disease-related confounder of body composition, they compared self-reported PA levels between groups. With a p-value of 0.052 their analysis almost reached significance for lower PA levels in patients. From a Swedish registry study including self-reported PA, Haglund [16] concluded that patients with spondyloarthritis are slightly more likely to meet PA recommendations than healthy controls and both groups exhibit sufficient PA (about 70%) in general. Cultural differences and over-reporting of PA in survey research may explain these inconsistencies with other studies on PA around the globe [25], [31]. Using a less sophisticated 3-axial accelerometer, Plasqui [15] found no differences in weekly PAL in a group of 25 patients with AS matched to healthy controls by gender, age and body mass. They observed a PAL (mean) between 1.70 and 1.99 indicating the selection of active or moderately active patients and controls, while our study sample can be classified as sedentary or light active with PAL values between 1.40 and 1.69 according to the World Health Organization guidelines on energy requirements [35]. In contrast to this work, the study of Plasqui [15] presented with a high risk of bias due to the small sample size (n = 25), the recruitment of first degree relatives as controls (about half the sample), no control for seasonal effects on PA and an inappropriate non-wear description (waking hours instead of 90% data of 24 hours period in this study). As we confirmed lower total PA with an objective methodology, we feel that research and maybe health policy on PA in aSpA should be prioritized.

This is the first study in the aSpA field that compared patients and controls across different PA intensity levels. We established a lack of weekly time spent at (very)vigorous PA and reduced MVPA, while only a trend for less moderate intensity PA between patients with aSpA and healthy controls was observed. The American College of Sports Medicine/American Heart association (ACSM/AHA) PA guideline recommends moderate intensity PA for a minimum of 30 min on five days each week or vigorous intensity PA for a minimum of 20 min on three days each week to maintain health [31]. Population surveys indicate that persons with self-reported doctor-diagnosed arthritis are less likely to meet PA guidelines for both moderate and vigorous activities (30 and 21%) compared to persons without arthritis (33 and 24%) [36]. We found that patients with aSpA and healthy controls spent on average 98 and 137 minutes (2.29 and 1.63 hrs) per day at moderate intensity and 0 and 4 minutes per day (0 and 0.07 hrs) at (very) vigorous PA. Both patients with aSpA and healthy controls appear to outperform the ACSM/AHA guideline for moderate, but not vigorous activities. Also, apparently sufficient levels but clinically relevant group differences in health enhancing MVPA were found (47 min less aSpA group). As this guideline only takes PA bouts of 10 minutes or more into account and allows combinations of moderate and vigorous activities to minimally accumulate 450 MET.min/week, direct comparison with our study data is impossible [31]. By including a control group, our study truthfully shows a disease-related loss of vigorous, very vigorous and moderate/(very)vigorous combined PA participation in patients with aSpA. Similarly, Farr [37] applied accelerometry in a sample of patients with osteoarthritis and observed a dramatic drop in patients who met the ACSM guideline for time spent at vigorous activities (men 2%, women 1%). In addition, the scarcity of time spent at vigorous and very vigorous intensity levels probably explains the differences in weekly average PAL and EE in patients versus controls. Last, although minimally clinically important difference estimates in aSpA do not exist for the PA estimates under study, the differences between groups exceeded measurement error [38] and are similar to treatment effects in other populations [39].

To date, PA studies in aSpA have mainly focused on the role of PA on other outcomes. Da Costa [14] concluded that higher doses of leisure time PA determined by a structured interview were associated with less fatigue severity in aSpA patients with a normal mental status, while this effect was absent for patients reporting a poor mental status as measured with the SF-36 health survey's mental component subscore. Ward [40] has identified high PA intensities at work as a predictor of structural damage and activity limitations in AS. The latter finding points to the question whether the observed reduction of time spent in vigorous and very vigorous activity levels is adaptive (i.e. protective to the underlying disorder) or maladaptive (i.e. compromising the underlying disorder or other health outcomes) in the context of aSpA [2], [41]. On one hand, a role for entheseal biomechanical stress in the development/maintenance of inflammation and/or damage in aSpA was recently proposed [2], [42]. The observed stable and lower levels of PA in the aSpA group may be an effective strategy to alleviate aSpA disease processes. Indeed, the most frequently reported arthritis-specific coping strategy by patients is changing PA in terms of duration, frequency and intensity to complete an ongoing task [43]. On the other hand, a relationship between PA and cardiovascular health exists [31] and refraining from (very) vigorous activities may add to the increased cardiovascular risk of patients with aSpA [3]. Thus, increasing PA without vast entheseal biomechanical stress may be of uttermost importance to optimize health-related physical fitness in these patients. A large body of evidence from intervention studies supports only moderate effects of exercise to improve pain, stiffness, mobility impairment, patient's global assessment and activity limitations [13]. Ince et al. [38] targeted energy expenditure in line with the ACSM/AHA guidelines to develop cardio-respiratory endurance patients with AS. Although core outcomes such as pain were not evaluated, the large effect sizes found for improvement in aerobic capacity point to rehabilitation opportunities in aSpA. Our finding that disease activity did not affect the observed differences between patients and healthy controls also suggests possibilities for PA intervention. Caution is however needed, because small but significant negative associations between total physical activity and both C-reactive protein [15] and BASDAI [16] were reported. In addition, no golden standard is available to assess disease activity and we only focused on self-reported disease activity. Future randomised studies including a wide spectrum of imaging, clinical (patient and physician perspective) and laboratory measures of disease activity may fully appreciate the role of disease activity. Also, as nor disease activity or work status, by intuition strong candidates to explain low PA, explained the PA differences observed, future research should focus on the identification of modifiable determinants of PA behaviour to promote health. For now, we interpret our finding as a non-recovery of reduced PA due to high disease activity, that needs a tailored rehabilitation approach beyond disease control.

The ASAS expert group recently embraced the World Health Organization's International Classification of Functioning, Disability and Health (WHO/ICF) framework to standardize the assessment of functioning in aSpA [44]. Unfortunately, the ASAS/WHO/ICF core sets [44]–[47] fail to recognize the crucial distinction between what a person can do in a standardized environment (activities) versus in a real-life situation (participation) [48]. More problematic is the continued use of self-reported outcome measures to appreciate functioning, possibly biased by psychological factors such as depression and anxiety [49]. This study adds to the optimal assessment of functioning in aSpA by quantifying the ‘amount of’ instead of ‘difficulty with’ movement-related participations and by introducing an unbiased objective measurement instrument in the patient's own environment.

The fact that our weekdays PA estimates were based on three instead of five weekdays may be considered as a limitation of this study. Because subjects were instructed to wear the SenseWear armband day and night, minimizing monitoring days based on a stability threshold established in healthy controls was needed to minimize patient burden without compromising validity [25], [50]. To our opinion, high levels of compliance, matching days and season, and the participant's similar cultural background has resulted in accurate measures of habitual PA in both groups. Also, in our exploratory part, pairwise comparisons between groups at each time point and the observed higher variability of PA in healthy controls across timepoints that may relate to different change profiles between groups did not turn out significant, possibly indicating a lack of power. Only the primary outcomes comparing weekly PA between groups and evaluating the role of disease activity can be confidently interpreted.

## Conclusions

This is the first study establishing differences in PA between patients with aSpA and healthy controls using objective multi-sensor PA measures. Major findings were reduced weekly average energy expenditure and time spent at vigorous, very vigorous and moderate/(very)vigorous combined PA in patients with aSpA. Interestingly, disease activity did not affect the observed disparities in PA. Therefore, unraveling the relationship between PA and clinical outcomes in patients with aSpA should be a research and maybe health policy priority.

## Supporting Information

Table S1 **Detailed comparison of physical activity parameters between healthy controls and patients with axial spondyloarthritis.**
(XLSX)
